# Everyday Cardiac Surgery in Jehovah‘s Witnesses of Typically Advanced Age: Clinical Outcome and Matched Comparison

**DOI:** 10.3390/jcm12155110

**Published:** 2023-08-03

**Authors:** Martin Hartrumpf, Ralf-Uwe Kuehnel, Roya Ostovar, Filip Schroeter, Johannes M. Albes

**Affiliations:** Department of Cardiovascular Surgery, Heart Center Brandenburg, University Hospital Brandenburg Medical School (Theodor Fontane), Ladeburger Strasse 17, 16321 Bernau bei Berlin, Germany; ralf-uwe.kuehnel@immanuelalbertinen.de (R.-U.K.); roya.ostovar@immanuelalbertinen.de (R.O.); filip.schroeter@immanuelalbertinen.de (F.S.); johannes.albes@immanuelalbertinen.de (J.M.A.)

**Keywords:** Jehovah’s Witnesses, cardiac surgery, outcome, transfusion, blood

## Abstract

Background and Objectives: Jehovah’s Witnesses (JW) reject the transfusion of blood components based on their religious beliefs, even if they are in danger of harm or death. In cardiac surgery, this significantly reduces the margin of safety and leads to ethical conflicts. Informed consent should be carefully documented and the patient’s family should be involved. This study aims to compare the postoperative course of JW who underwent major cardiac surgery with a similar population of non-Witnesses (NW). Patients and Methods: Demographic, procedural, and postoperative data of all consecutive JW who underwent cardiac surgery at our institution were obtained from the records. They were compared with a propensity-score-matched group of NW. Anemic JW were treated with erythropoietin and/or iron as needed. Cardiac surgery was performed by experienced surgeons using median sternotomy and cardiopulmonary bypass. Common blood-sparing techniques were routinely used. Periprocedural morbidity and mortality were statistically evaluated for both groups. Results: A total of 32 JW and 64 NW were part of the matched dataset, showing no demographic or procedural differences. EPO was used preoperatively in 34.4% and postoperatively in 15.6% of JW but not in NW. Preoperative hemoglobin levels were similar (JW, 8.09 ± 0.99 mmol/L; NW, 8.18 ± 1.06; *p* = 0.683). JW did not receive any transfusions except for one who revoked, while NW transfusion rates were 2.5 ± 3.1 units for red cells (*p* < 0.001) and 0.3 ± 0.8 for platelets (*p* = 0.018). Postoperative levels differed significantly for hemoglobin (JW, 6.05 ± 1.00 mmol/L; NW, 6.88 ± 0.87; *p* < 0.001), and hematocrit (JW, 0.29 ± 0.04; NW, 0.33 ± 0.04; *p* < 0.001) but not for creatinine. Early mortality was similar (JW, 6.3%; NW, 4.7%; *p* = 0.745). There were more pacemakers and pneumonias in JW, while all other postoperative conditions were not different. Conclusions: Real-world data indicate that Jehovah’s Witnesses can safely undergo cardiac surgery provided that patients are preconditioned and treated by experienced surgeons who use blood-saving strategies. Postoperative anemia is observed but does not translate into a worse clinical outcome. This is consistent with other studies. Finally, the results of this study suggest that all patients should benefit from optimal pretreatment and blood-sparing strategies in cardiac surgery, not just Jehovah’s Witnesses.

## 1. Introduction

Jehovah’s Witnesses object to the transfusion of blood and blood components because of their religious beliefs. Specifically, this involves the administration of red blood cell concentrates, fresh plasma, platelets, or white blood cells. They do so in accordance with their right to self-determination, even if refusal would result in physical harm or death [1,2,3,4,5,6]. Predonation of autologous blood is also refused, as blood must not leave the body to remain clean, according to their beliefs [7,8]. In contrast, cell salvage and autologous retransfusion are generally accepted as long as the blood circulates in a closed loop and thus remains in continuity with the body [3,8]. As a rule, corresponding declarations of intent are submitted in written form and contain detailed information about the products and methods that are accepted or rejected [2,9].

Surgery without the use of blood products is a challenge for the surgeon in charge. Every surgeon strives to operate in a blood-saving manner, but unforeseen situations can always arise. In this case, blood loss cannot be compensated for and impaired coagulation can only be treated with restrictions. Taken together, this reduces the margin of safety and can lead to ethical conflicts between the patient and the treating physicians. This is particularly true when minors or other vulnerable individuals are involved. To date, there are no strict guidelines and many clinics do not want to take this risk and therefore refuse such patients. Furthermore, no physician can be forced to provide treatment if it creates ethical conflicts.

The patient and family must be thoroughly informed about blood-sparing treatment options and the consequences of not receiving a transfusion. If this is a realistic possibility, they should be explicitly informed about permanent damage or death. Another concern in this regard is the management of patients who are already anemic. Jehovah’s Witnesses usually provide pre-printed forms that explicitly document refusal of blood transfusion. It should be clarified in detail which blood components and procedures are acceptable and which are not [2,10]. All discussions should be carefully documented and the results should be countersigned in the presence of a witness, if appropriate. However, the patient’s will should be reassessed throughout the course of treatment, especially if the situation becomes critical [11].

In general, it is known from previous studies that clinical outcomes after cardiac surgery in Jehovah’s Witnesses (JW) are largely similar to those in non-Witnesses (NW). Chambault [7], in a review of 11 comparative studies, was able to show that in-hospital mortality rates were not different between JW (ranging from 0 to 18.8%) and NW (0 to 8.5%). Even urgent surgery could be performed at low risk, despite a lack of preoptimization of Hb levels. Rates of acute myocardial infarction (ranging from 0 to 6.3% in JW and 0 to 4.2% in NW) and stroke (0–5.1% in both groups) or infections were not statistically different. Results for acute kidney injury varied widely among studies but showed no overall differences. Some studies reported shorter length of stay in the hospital for witnesses, and one study even reported a shorter stay in the intensive care unit. One study reported on long-term survival of JW and showed better survival rates at 5, 10, 15, and 20 years after surgery compared to NW. Quality of life was similar. Vasques [12] analyzed six studies with comparable groups. JW had higher Hb levels before and after cardiac surgery (postoperative data from four studies: 11.5 g/L vs. 9.8 g/L; *p* < 0.001) and had significantly less postoperative blood loss (402 mL vs. 826 mL; *p* < 0.001). Early mortality was not different (pooled rates: 2.6% vs. 3.6%; *p* = 0.318). There were also no differences in atrial fibrillation, stroke, myocardial infarction, reoperation for bleeding, acute kidney injury, and length of ICU stay. Marinakis [13] focused on laboratory results and found that hemoglobin, platelet, and serum creatinine levels did not differ at different time points pre- and postoperatively. None of the JW but 27.4% of the NW controls received red-blood-cell transfusions with a mean of 0.63 ± 1.5 units. Perioperative parameters (ECC time, clamp time, blood loss, drainage performance) and postoperative outcome (mediastinitis, hemorrhage, tamponade, LVEF, renal damage, stroke, in-hospital mortality) did not differ between groups. In conclusion, previous studies suggest that cardiac surgery in Jehovah’s Witnesses does not carry an increased risk of morbidity and mortality, provided that optimal pretreatment and a bloodless protocol are used. In fact, it has been suggested that such an approach may offer benefits to all patients undergoing cardiac surgery, not just Jehovah’s Witnesses.

As of 2022, there are nearly 8.7 million Jehovah’s Witnesses in 239 countries worldwide, according to their website [14]. Jehovah’s Witnesses are occasionally encountered in a cardiac surgery unit. They may even be older and frailer than the standard population. The purpose of this retrospective observational study was to compare the clinical outcomes of Jehovah’s Witnesses undergoing cardiac surgery at our institution with those of non-Witnesses. Propensity score matching was required to adjust for differences in patient characteristics.

## 2. Patients and Methods

### 2.1. Study Design

We retrospectively analyzed the medical records of all consecutive Jehovah’s Witnesses who underwent major cardiac surgery at our institution between 2003 and 2022. The study was conducted as an all-comers observational study with no specific inclusion criteria or protocols. Data were completely anonymized so that written informed consent was not required. The study was approved by our institutional ethics committee (No. E-01-20230411). Demographic characteristics, procedural data, and short-term postoperative outcomes were obtained from patient records.

### 2.2. Surgical Management

In all elective patients, anticoagulants and antiplatelet agents, except acetylsalicylic acid, were discontinued preoperatively in a timely manner and replaced with bridging therapy as needed. Jehovah’s Witnesses received erythropoietin and/or iron preoperatively only in cases of relevant anemia (i.e., hemoglobin < 8 mmol/L, hematocrit < 40%), although there was no specific protocol for this. In this case, epoetin alfa was administered 3 times per week at a dose of 50 IU/kg. In case of iron deficiency, EPO was accompanied by oral iron (Fe^2+^ 100–200 mg per day) until hemoglobin reached acceptable levels. This treatment was administered after admission to the hospital and surgery was postponed for 1–2 weeks. All other patients did not receive EPO.

All patients underwent standard cardiac surgery. Only a few surgeons with the highest level of experience were involved. Access was obtained through a median sternotomy. Cardiopulmonary bypass was performed in all cases, with cannulation and temperature level as needed. We did not use a dedicated minimally invasive extracorporeal circuit. However, retrograde autologous priming and intraoperative ultrafiltration for hemoconcentration were routinely used in Jehovah’s Witnesses. No specific surgical strategy was used in Jehovah’s Witnesses other than general blood-sparing techniques, including meticulous hemostasis and squeezing of all surgical swabs. The administration of blood derivatives such as clotting factors or local hemostatic agents and cell salvage were permitted in most cases. However, autologous blood collection (hypovolemic hemodilution) was generally not an option. In all cases, strategies strictly adhered to the individual patient’s written decision. Postoperative blood sampling was kept to a minimum.

### 2.3. Statistical Methods

Statistics were calculated using IBM SPSS 23 (IBM, Armonk, NY, USA) and R 4.2.2 (R Core Team 2022. R: A language and environment for statistical computing. R Foundation for Statistical Computing, Vienna, Austria. URL https://www.R-project.org/, accessed on 29 December 2022).

Continuous values are given as mean ± standard deviation as well as median with interquartile range (IQR), categorical values as percentages and their counts in parentheses. Tests for normal distribution were performed with SPSS. The Kolmogorov–Smirnov test is used for larger sample sizes (>50) but becomes significant for exceptionally large samples, which is true for our dataset. Visual evaluation of Q-Q plots was preferred here. The Shapiro–Wilk test is appropriate for smaller sample sizes and was therefore used for the matched dataset. The qualitative result for the normality test is given in the tables for each continuous parameter. Differences between means were tested using either Student’s independent *t*-test or Mann–Whitney’s U-Test, depending on normality. In addition to the conventional *p*-values, the standard mean differences (SMDs), also known as Cohen’s d, are also reported for continuous values to provide a better estimate of the effect size. Common evaluation limits are as follows: 0 = no effect, 0.2 = small effect, 0.5 = medium effect, 0.8 = large effect. Categorical values were tested for differences using the Chi-square test. Paired values (before–after) were tested with the dependent *t*-test or Wilcoxon’s test, depending on normality. R was used with the “MatchIt”-Package (version 4.5.1) for propensity score matching.

## 3. Results

By June 2022, there were 32 Jehovah’s Witnesses (JW) and 24,285 non-Witnesses (NW). The dataset was not only extremely unbalanced, but also highly disparate in terms of gender, weight, and BMI. In particular, the gender distribution in the small group of Jehovah’s Witnesses was almost even, while men predominated in the general cardiosurgical population. In addition, the JW patients were significantly less obese (Table 1).

Two-step propensity score matching was performed to adjust for this difference. In the first step, a 3:1 matching was performed on age, gender, BMI, urgency, and logistic EuroSCORE because these values were consistently available in the past. Missing information such as EuroSCORE II, procedure times, laboratory results, and medical history were then obtained and added to the contracted dataset. In the second step, final 2:1 propensity score matching was performed for age, gender, BMI, EuroSCORE II, repeat surgery, temperature, and duration of surgery. The matched dataset showed no further significant differences in body measurements, risk profiles, and spectrum of pre-existing conditions (Table 2). Brief definitions of the clinical conditions are available in the Supplementary materials. In the matched dataset, eight different surgeons were involved in the JW group (including the head of department in 20 cases) and 18 different surgeons in the NW group.

The procedural data are presented in Table 3. EPO was administered to about one-third of the Jehovah’s Witnesses but not to other patients. Surgical procedures were evenly distributed between the groups. Due to the large number of combined procedures, the proportion of surgical procedures (valves, CABG, aortic, arch, and others) amount to more than 100% in both groups. Notably, there was a considerable proportion of ascending aortic and arch surgeries in the JW group without reaching statistical significance. Data on blood loss were not available. Finally, there were no differences in lowest temperature, length of stay, or surgery-related times.

Table 4 shows the short-term clinical outcomes. Almost all postoperative conditions occurred at statistically similar rates, with the exception of pneumonia, which was clinically more common in the Jehovah’s Witnesses. However, pre-discharge hemoglobin and hematocrit levels were significantly lower in the JW group. In the NW group, 40 patients (62.5%) received red-blood-cell (RBC) concentrates and 24 patients (37.5%) did not. Of those who received RBCs, the range was 1 to 18 with a median of 2. Ten patients (15.6%) in the NW group received platelets (range, 2 to 4; median, 2), while 54 patients (84.4%) did not. One in six Jehovah’s Witnesses received EPO during the postoperative course but none of the non-Witnesses did. As Jehovah’s Witnesses did not receive blood components, there was a strong statistical difference with the NW group in the administration of red blood cells and platelets. The only exception was one patient who revoked and allowed a postoperative transfusion of four red-cell concentrates due to severe anemia. Notably, there was no difference in in-hospital mortality.

**Figure 1:**
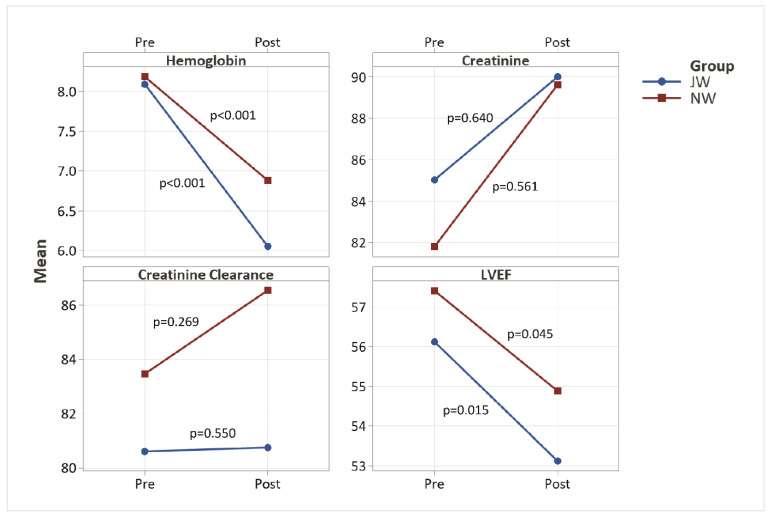
Perioperative changes of mean levels according to patient groups. Blue, Jehovah’s Witnesses (JW); red, non-Witnesses (NW). Upper left, hemoglobin (Hb) (mmol/L); upper right, creatinine (µmol/L); lower left, creatinine clearance (mL/min); lower right, LVEF (%). The corresponding *p*-values of the paired changes are also shown. The changes are significant for hemoglobin, hematocrit (not shown) and LVEF, but not for creatinine and its clearance.

Figure 1 shows the changes of hemoglobin, creatinine, creatinine clearance, and LVEF grouped by Jehovah’s Witnesses and non-Witnesses. For both groups (JW, NW), the changes were significant for LVEF, hemoglobin, and hematocrit (not shown) but not significant for creatinine and its clearance. While the perioperative changes for hemoglobin and hematocrit are highly relevant, the small decrease in LVEF appears clinically negligible despite its statistical significance. However, this does not affect the overall conclusion.

## 4. Discussion

Our current study compared 32 consecutive Jehovah’s Witnesses undergoing cardiac surgery with a matched cohort of non-Witnesses. Although the sample size appears relatively small, it is consistent with similar group sizes in other published studies [9,13,15,16,17]. The small cohort was characterized by a nearly balanced gender ratio and below-average weight and BMI. This was in contrast to the dominance of the male gender and the elevated BMI in the non-Witness cohort, and thus required statistical adjustment. The reason for this is unclear. Jehovah’s Witnesses do not have specific dietary requirements. However, they practice a Bible-compliant lifestyle that avoids impure or unhealthy behaviors. They do not eat foods containing blood, some are vegetarian, and alcohol is allowed only in moderation. Smoking and drug use are banned [6]. In this context, they presented with less hypertension, less smoking and fewer myocardial infarctions in their history, although this did not reach statistical significance in the matched cohort.

We demonstrated that Jehovah’s Witnesses had significantly lower postoperative hemoglobin and hematocrit levels due to their refusal of blood transfusions. The perioperative decrease in hemoglobin and hematocrit was more pronounced in Jehovah’s Witnesses. Accordingly, there was a significant statistical difference in the transfusion rate between the two groups. Instead, Jehovah’s Witnesses received erythropoietin postoperatively in case of unacceptable anemia. Although only a symptomatic treatment for anemia, the benefit for surgical patients is well known, and administration of EPO is recommended by the 2017 EACTS guidelines especially in the preoperative setting [18]. There are advanced protocols for the treatment of anemic patients that show gradual improvement as early as 1–2 weeks after initiation [2,3,4,10,19,20,21]. This should not only apply to Jehovah’s Witnesses, but should be generally practiced in all anemic patients [22]. A target hemoglobin of >12 g/dL by elective administration of erythropoietin or iron has been shown to improve both morbidity and mortality during cardiovascular surgery in JW patients [21].

Notably, refusal of blood transfusion was not associated with worse clinical outcomes. This is consistent with a number of other studies [7,13,15,23,24,25]. In particular, our in-hospital mortality was not different between the two groups. There was also no difference in rethoracotomy or pericardiocentesis or in other common endpoints such as atrial fibrillation, myocardial infarction, renal failure, sepsis, stroke, or delirium. The only detectable difference was a higher incidence of pneumonia and new pacemaker implantation in the JW group. As an exception to the rule, we had one JW patient who allowed a transfusion of red-blood-cell concentrates when he/she was not progressing in the healing process due to his/her poor general condition.

Other studies have also shown that Jehovah’s Witnesses are not at higher risk for in-hospital adverse events or mortality, and do not show impaired long-term survival compared to non-Witnesses, unless they are severely anemic (<8 g/dL) in the postoperative course [23]. In this case, the mortality in the JW group reached up to 40%. We could not verify this in our patient cohort. Other groups have reported in-hospital mortality of 2.9–5.0% in Jehovah’s Witnesses [9,13,15]. A pooled analysis also found an in-hospital mortality rate of 2.6% and a non-significant trend toward lower rates of stroke, myocardial infarction, atrial fibrillation, reoperation for bleeding, and shorter ICU stay [12]. In our cohort, we observed a slightly higher mortality rate in both groups compared to other studies. This may be related to the more complex and bleeding-prone procedures in our JW, which involved replacement of the ascending aorta or aortic arch in 21.9% of cases, endocarditis in 12.5%, redo surgery in 6.7%, and a logistic EuroSCORE of 10.18 ± 14.91%. In comparison, Müller et al. reported 5.8% aortic procedures, 2.9% endocarditis, and a logistic EuroSCORE of 6.93 ± 7.51% [15]. The pooled analysis of Vasques et al. reported only 3.2% of other major procedures (non-CABG and non-valve) and no explicit endocarditis or redo cases [12]. In the study of Marinakis, the rate of aortic interventions was 6%, redo cases 10%, and no endocarditis was reported [13]. Moreover, some previous studies presented JW patients aged 62 to 64 years [12,13,16,17], while our Witnesses averaged 68.1 ± 9.4 years of age. Although not really geriatric patients, this may reflect an increasingly aging society. This is not unique to Jehovah’s Witnesses. However, since the risk of surgery is directly related to age, the indication for bloodless surgery must be even more narrowly defined.

Furthermore, long-term survival is not different from that of the standard patient population. Wauthy et al. demonstrated a median survival after cardiac surgery of 21.1 years in Jehovah’s Witnesses and 20.3 years in the control group (*p* = 0.37) [16]. Quality of life is also unaffected based on questionnaires in terms of physical, emotional, social and global scores, as shown by the same Belgian research group [16]. However, we did not evaluate long-term outcomes in our patients.

Our pre-treatment with EPO was similar to other groups and included only one-third of all patients presenting with unacceptable hemoglobin levels. In our previous approach, such treatment was considered costly and time-consuming, while at the same time many patients were considered urgent. For the same reason, outpatient treatment with EPO was not available. Similarly, Müller et al. reported a rate of 34.3% of EPO administration in a group of 35 JW, resulting in an average hemoglobin increase of 2.0 g/dL [15]. Their patients presented with hemoglobin levels on admission similar to ours (JW, 14.1 ± 1.1 vs. 13.0 ± 1.6 g/dL; non-Witnesses, 13.2 ± 2.0 vs. 13.2 ± 1.7 g/dL). However, their Witnesses had significantly higher postoperative hemoglobin than the non-Witnesses (11.5 ± 1.5 vs. 10.3 ± 1.3 g/dL, *p* < 0.001), even though the latter were transfused. The authors attributed this to the high efficacy of multimodal blood-conservation strategies. Higher postoperative Hb levels in Jehovah’s Witnesses were also shown in a meta-analysis (11.5 g/dL vs. 9.8 g/dL, *p* < 0.001) [12] and in a large Australian database (10.8 ± 1.5 vs. 9.9 ± 1.2 g/dL, *p* = 0.003) [26]. Other studies showed no postoperative Hb differences between groups (10.7 ± 2.5 vs. 11.4 ± 1.8 g/dL) [13]. However, this could not be confirmed in our study, where JW patients had significantly lower postoperative hemoglobin (9.8 ± 1.6 vs. 11.1 ± 1.4 g/dL, *p* < 0.001). We treated only one sixth of all JW with severe postoperative anemia and were in poor condition, while most of them could be discharged in time. The study reflects our daily practice in the real world, without strict application of defined optimization protocols. However, recent evidence suggests that EPO treatment of anemic patients undergoing cardiac surgery may be cost-effective and effective even in the very short term. In a large randomized controlled trial reported by Spahn et al. [27], the administration of intravenous iron, subcutaneous erythropoietin alpha, vitamin B12, and oral folic acid on the day before cardiac surgery to anemic patients reduced the need for red-blood-cell concentrates and total-blood-product transfusions. This suggests that even ultrashort pretreatment of such patients exerts a sustained hematopoietic effect beyond surgery. Anemia treatment should therefore be extended to emergency patients, as there is no need to delay surgery.

Transfusions are generally known to have adverse effects in cardiac surgery patients [22]. Patients with advanced age, high EuroSCORE, low preoperative hemoglobin, combined surgery, and prolonged surgery are associated with higher transfusion rates. These patients have more than three times the risk of early mortality [28]. Octogenarians are particularly at risk after cardiac surgery. Transfusion of more than two units of red blood cells significantly increases their postoperative mortality. It also significantly prolongs their stay in the intensive care unit and in the hospital [29]. In addition, red blood cell transfusion increases the risk of infection [30] and stroke [31] after cardiac surgery. Platelet transfusion, in contrast, has not been identified as a risk factor for morbidity in such patients [32]. For the above reasons, a blood-sparing strategy and avoidance of transfusion seems generally advisable for all cardiac-surgery patients. In view of the scarcity of blood and the high price of blood products, cost considerations also support this approach [33]. The implementation of a dedicated blood management program not only leads to a reduced use of blood products and cost savings, but even improves the patient’s outcome [34]. A restrictive transfusion strategy has been found to be non-inferior to a liberal strategy in terms of morbidity and mortality in intermediate- to high-risk cardiac surgery patients [35]. Some authors have raised the question why this should apply only to Jehovah’s Witnesses and not to all patients [17]. Our transfusion policy has become more restrictive over the years based on recent literature. Currently, we correct preoperative anemia in non-Witnesses only in severe cases (<6 mmol/L) by administering 1–2 red-blood-cell concentrates. Intraoperative autologous blood transfusion is always preferred. In cases of prolonged ECC, severe hemodilution, or external blood loss, an appropriate number of RBC units are administered before discontinuing ECC. Postoperative anemia below a critical cut-off also leads to transfusion in symptomatic patients (<6 mmol/L) or when absolutely indicated (<4.5 mmol/L). Platelets are ordered early for thrombocytopathy, thrombocytopenia (<50,000/µL), aortic surgery, or emergent cases on antiplatelet therapy. Short-term platelet administration is reasonable in cases of unexpectedly extensive surgery, prolonged ECC time, von Willebrand disease, or significant bleeding despite the administration of other agents.

A useful summary of alternatives to transfusion can be found in the articles by Chambault [7] or Crowe [10]. There are also powerful tools for postoperative coagulation management such as ROTEM [17]. As a future perspective, there are new developments in the field of synthetic blood alternatives and hemoglobin-based oxygen carriers to overcome the religious problem of conventional blood transfusion [36].

In our experience, it is important to respect the patient’s wishes, discuss blood-sparing strategies on an individual basis, present alternatives, and involve the family in the process. Three final considerations are on the table: First, given the identical outcomes in both groups, why do the non-JW patients receive blood products at all? And, second, if there is an additional risk in not transfusing, is it outweighed by the surgeon’s experience? Ultimately, does this even mean that all procedures should be performed without blood in all patients and only by experienced surgeons? Certainly, a controversial question. Last but not least, the principle of medical action “Primum non nocere, secundum cavere, tertium sanare” should apply to all patients without exception.

### Limitations

The present study has the known limitations of a retrospective, single-center study. The group size is relatively small given the sporadic occurrence of such patients. In addition, selection bias may have occurred as the subgroup of Jehovah’s Witnesses was treated by the most experienced surgeons and probably received more attention in terms of blood-sparing strategies, anemia management, optimized coagulation treatment, and expedited surgery. This fact has also been recognized by other authors [17].

## 5. Conclusions

Our real-world data suggest that Jehovah’s Witnesses can safely undergo bloodless surgery without compromising clinical outcomes. Reduced hemoglobin levels were mostly well tolerated without affecting morbidity and mortality. This is in agreement with other studies. Given the limited number of patients available worldwide, we believe that our data contribute to the existing evidence. Finally, the results of this study suggest that all patients should benefit from optimal pretreatment and blood-sparing strategies in cardiac surgery, not just Jehovah’s Witnesses.

## Figures and Tables

**Table 1:** Unmatched preoperative data (raw data).

		Norm	JW n = 32	NW n > 20,000	*p* Value	SMD
Age	years	+	68.1 ± 9.4 69.0 (62.8–74.8)	67.6 ± 10.0 69.0 (61.0–75.0)	0.786	−0.048
Gender	male		53.1% (17)	72.4% (17,571)	0.015	
Height	cm	+	169 ± 9 170 (163–176)	171 ± 9 171 (165–177)	0.200	0.227
Weight	kg	+	76 ± 14 78 (64–88)	83 ± 16 82 (72–92)	0.011	0.452
BMI	kg/m^2^	-	26.43 ± 4.73 25.14 (23.07–29.57)	28.50 ± 4.82 27.92 (25.15–31.22)	0.005	0.431
BSA	m^2^	+	1.88 ± 0.21 1.93 (1.73–2.03)	1.81 ± 0.59 1.95 (1.79–2.10)	0.510	−0.116
log ES	%	-	10.25 ± 14.89 6.73 (2.87–11.97)	10.50 ± 14.79 5.02 (2.27–11.58)	0.293	0.017
Emergency			9.4% (3)	9.2% (2236)	0.976	
Redo			6.7% (2)	4.4% (1072)	0.549	

JW, Jehovah’s Witnesses; NW, non-Witnesses. Continuous values are shown as mean ± SD as well as median and interquartile range (IQR), categorical values as percentages and count. *p* values below 0.05 were considered significant. BMI, body mass index; BSA, body surface area; log ES, logistic EuroSCORE; Norm, normal distribution; SMD, standard mean difference.

**Table 2:** Patient characteristics (propensity-score-matched dataset).

		Norm	JW n = 32	NW n = 64	*p* Value	SMD
Age	years	-	68.1 ± 9.4 68.5 (61.0–74.0)	67.4 ± 9.6 69.0 (62.8–74.8)	0.735	0.074
Gender	male		53.1% (17)	57.8% (37)	0.663	
Height	cm	+	168 ± 9 169 (160–178)	168 ± 12 169 (163–174)	0.995	0.001
Weight	kg	+	76 ± 14 78 (66–85)	77 ± 15 78 (64–88)	0.772	0.063
BMI	kg/m^2^	+	26.84 ± 4.79 26.08 (24.08–31.24)	27.16 ± 4.59 26.37 (23.07–29.79)	0.746	0.070
BSA	m^2^	+	1.86 ± 0.19 1.88 (1.69–2.02)	1.87 ± 0.23 1.90 (1.73–1.99)	0.811	0.052
log ES	%	-	10.18 ± 14.91 4.74 (2.58–8.13)	8.07 ± 10.92 6.51 (2.87–11.97)	0.214	−0.171
ES II	%	-	7.59 ± 15.97 2.08 (1.42–3.55)	5.30 ± 11.24 2.97 (1.61–6.56)	0.087	−0.176
LVEF preop	%	-	56 ± 11 60.0 (50.0–60.0)	57 ± 11 58.5 (50.0–61.0)	0.749	0.118
Emergency			9.4% (3)	6.3% (4)	0.579	
Redo surgery			6.3% (2)	7.8% (5)	0.781	
Hemoglobin	mmol/L	+	8.09 ± 0.99 8.30 (7.55–9.00)	8.18 ± 1.06 8.10 (7.60–8.75)	0.683	0.089
	g/dL		13.04 ± 1.60 13.37 (12.16–14.49)	13.18 ± 1.71 13.04 (12.24–14.09)		
Hematocrit		+	0.39 ± 0.05 0.39 (0.36–0.43)	0.39 ± 0.05 0.38 (0.35–0.42)	0.645	0.100
Creatinine	µmol/L	+	85.0 ± 21.6 79.50 (70.00–97.00)	81.8 ± 18.6 79.50 (72.25–103.50)	0.452	−0.164
Crea Clearance	mL/min	-	80.6 ± 39.7 77.74 (64.41–96.80)	83.5 ± 31.9 78.64 (53.38–90.98)	0.499	0.082
Endocarditis			12.5% (4)	3.1% (2)	0.074	
Malignant disease			3.1% (1)	4.7% (3)	0.718	
Arterial hypertension			59.4% (19)	78.1% (50)	0.054	
Hyperlipidemia			50.0% (16)	46.9% (30)	0.773	
Smoking history			3.1% (1)	14.1% (9)	0.098	
NIDDM			12.5% (4)	25.0% (16)	0.155	
IDDM			9.4% (3)	9.4% (6)	1.000	
Stroke			3.1% (1)	1.6% (1)	0.613	
PVD			6.3% (2)	10.9% (7)	0.458	
CVD			3.1% (1)	3.1% (2)	1.000	
Myocardial infarction			9.4% (3)	25.0% (16)	0.070	
COPD			9.4% (3)	9.4% (6)	1.000	
Atrial fibrillation			25.0% (8)	10.9% (7)	0.074	
Pacemaker			3.1% (1)	6.3% (4)	0.516	

JW, Jehovah’s Witnesses; NW, non-Witnesses. Continuous values are shown as mean ± SD as well as median and interquartile range (IQR), categorical values as percentages and count. *p* values below 0.05 were considered significant. BMI, body mass index; BSA, body surface area; COPD, chronic obstructive pulmonary disease; CVD, cerebrovascular disease; ES II, EuroSCORE II; log ES, logistic EuroSCORE; LVEF, left ventricular ejection fraction; PVD, peripheral vascular disease; Norm, normal distribution; SMD, standard mean difference.

**Table 3:** Perioperative data (propensity-score-matched dataset).

		Norm	JW n = 32	NW n = 64	*p* Value	SMD
Preoperative EPO			34.4% (11)	0.0% (0)	0.006	
Valve surgery			56.3% (18)	48.4% (31)	0.470	
CABG			56.3% (18)	67.2% (43)	0.294	
Ascending aorta			15.6% (5)	4.7% (3)	0.068	
Aortic arch			6.3% (2)	1.6% (1)	0.213	
Other cardiac procedures			9.4% (3)	10.9% (7)	0.813	
Temperature	°C	-	34.1 ± 3.3 35 (34–36)	34.3 ± 3.0 36 (32–36)	0.444	0.070
Duration of surgery	min	-	226 ± 74 225 (170–277)	227 ± 88 209 (166–263)	0.661	0.011
ECC time	min	-	132 ± 64 138 (89–173)	124 ± 60 112 (90–139)	0.341	−0.123
Clamp time	min	-	81 ± 40 82 (57–105)	73 ± 32 66 (55–81)	0.159	−0.227
Total length of stay	days	-	20.8 ± 10.1 20.0 (12.5–26.8)	18.4 ± 8.2 15.6 (12.3–23.2)	0.343	−0.268
ICU length of stay	days	-	4.0 ± 6.5 1.0 (0.9–1.9)	3.0 ± 6.5 1.0 (0.7–2.3)	0.252	−0.145
Postop length of stay	days	-	16.4 ± 8.4 14.8 (10.2–22.8)	14.7 ± 7.5 13.0 (9.7–17.0)	0.351	−0.227

JW, Jehovah’s Witnesses; NW, non-Witnesses. Continuous values are shown as mean ± SD as well as median and interquartile range (IQR), categorical values as percentages and count. *p* values below 0.05 were considered significant. CABG, coronary artery bypass grafting; ECC, extracorporeal circulation; EPO, erythropoietin; ICU, intensive care unit; Norm, normal distribution; SMD, standard mean difference.

**Table 4:** Postoperative data (propensity-score-matched dataset).

		Norm	JW n = 32	NW n = 64	*p* Value	SMD
LVEF postop	%	-	53 ± 11 60.0 (46.3–60.0)	55 ± 11 60.0 (50.0–60.0)	0.521	0.166
Hemoglobin	mmol/L	+	6.05 ± 1.00 6.10 (5.43–6.78)	6.88 ± 0.87 6.75 (6.20–7.40)	<0.001	0.903
	g/dL		9.75 ± 1.61 9.82 (8.74–10.92)	11.09 ± 1.40 10.87 (9.98–11.92)		
Hematocrit		+	0.29 ± 0.04 0.30 (0.26–0.32)	0.33 ± 0.04 0.32 (0.30–0.36)	<0.001	0.767
Creatinine	µmol/L	-	90.0 ± 41.3 78.00 (68.75–95.75)	89.6 ± 70.7 80.00 (60.00–94.00)	0.614	−0.006
Crea Clearance	mL/min	-	80.8 ± 34.6 76.83 (56.60–102.07)	86.5 ± 38.2 79.01 (57.57–105.91)	0.603	0.156
Red blood cell administration	units	-	0.1 ± 0.7 0 (0)	2.5 ± 3.1 2 (0–4)	<0.001	0.948
Platelet administration	units	-	0.0 ± 0.0 0 (0)	0.3 ± 0.8 0 (0)	0.018	0.506
Postoperative EPO			15.6% (5)	0.0% (0)	0.001	
Rethoracotomy (bleeding, tamponade)			6.3% (2)	6.3% (4)	1.000	
Pericardiocentesis			6.3% (2)	1.6% (1)	0.213	
New onset atrial fibrillation			12.5% (4)	21.9% (14)	0.267	
New pacemaker			6.3% (2)	0.0% (0)	0.043	
Myocardial infarction			0.0% (0)	1.6% (1)	0.477	
Wound healing disorders			6.3% (2)	7.8% (5)	0.781	
New onset dialysis			9.4% (3)	3.1% (2)	0.194	
Pneumonia			6.3% (2)	0.0% (0)	0.043	
Tracheostomy			6.3% (2)	3.1% (2)	0.470	
Septicemia			0.0% (0)	3.1% (2)	0.312	
Stroke			0.0% (0)	1.6% (1)	0.477	
Delirium			18.8% (6)	20.3% (13)	0.856	
Early Mortality			6.3% (2)	4.7% (3)	0.745	

JW, Jehovah’s Witnesses; NW, non-Witnesses. Continuous values are shown as mean ± SD as well as median and interquartile range (IQR), categorical values as percentages and count. *p* values below 0.05 were considered significant. EPO, erythropoietin; LVEF, left ventricular ejection fraction; Norm, normal distribution; SMD, standard mean difference.

## Data Availability

The data underlying this article are available in the article and in Supplementary Materials.

## Supplementary Materials

The following supporting information can be downloaded at: https://www.mdpi.com/article/10.3390/jcm12155110/s1.
