# Supplementary material for: Everyday Cardiac Surgery in Jehovah‘s Witnesses of Typically Advanced Age: Clinical Outcome and Matched Comparison
Source: J Clin Med. 2023 Aug 3;12(15):5110. doi: 10.3390/jcm12155110 (PMC10420128; doi:10.3390/jcm12155110)
Supplement: Supplementary file 1 [file jcm-12-05110-s001.zip › Clinical _definitions.pdf]

Brief definitions of the clinical parameters used in the study

The definitions of conditions used in table 2 are either commonplace or based on those of EuroSCORE as follows:

Endocarditis is defined as active infective endocarditis (meeting Duke criteria) requiring surgery due to increased risk of embolism, heart failure, valve destruction, abscess formation, or persistent infection. Patients are treated with antibiotics at the time of surgery.

Malignant disease is present when the patient has a malignant neoplasm that limits life expectancy.

Arterial hypertension is defined according to the European Society of Cardiology, when blood pressure exceeds 140/90 mmHg at rest.

Hyperlipidemia refers to increased levels of total cholesterol (>5.2 mmol/l), LDL cholesterol (>3 mmol/l), or triglycerides (>2.3 mmol/l).

NIDDM and IDDM differ by current use of insulin at the time of admission.

Stroke is defined as a preceding ischemic neurologic event with or without permanent deficits.

Peripheral, cerebrovascular, or coronary artery disease follow the common definitions.

The **COPD** definition is linked to the administration of steroids or bronchodilators.

Atrial fibrillation is counted if this disorder is present in chronic intermittent or permanent form at the time of admission.

Pacemaker carrier status applies to any pacemaker or ICD already in place prior to surgery, thus excluding the same patient from counting for postoperative pacemaker implantation.
